# Level of adherence to dietary recommendations and barriers among type 2 diabetic patients: a cross-sectional study in an Ethiopian hospital

**DOI:** 10.1186/s40842-018-0070-7

**Published:** 2018-11-29

**Authors:** Asnakew Achaw Ayele, Yohannes Kelifa Emiru, Sofonyas Abebaw Tiruneh, Belete Achamyelew Ayele, Alemayehu Digssie Gebremariam, Henok Getachew Tegegn

**Affiliations:** 1 Department of Clinical Pharmacy, School of Pharmacy, College of Medicine and Health Science, University of Gondar, BOX 520 Gondar, PO Ethiopia; 2 School of Pharmacy, College of Medicine and Health Science, University of Gondar, Gondar, Ethiopia; 3 Department of Public Health, College of Health Sciences, Debre Tabor University, Debre Tabor, Ethiopia; 4 Department of Epidemiology and Biostatics, Institute of Public health College of Medicine and Health Science, University of Gondar, Gondar, Ethiopia; 5 School of Medicine and Pharmacy, University of New England, Armidale, NSW 2351 Australia

**Keywords:** Type 2 diabetes, Diabetic, Dietary recommendations, Adherence, Ethiopia

## Abstract

**Background:**

Limited data are available regarding the level of adherence and barriers to dietary recommendations in individuals with type 2 diabetes in Africa including Ethiopia. Therefore, this study aimed at assessing the level of dietary adherence and its barriers among patients with type 2 diabetes in northwest Ethiopia.

**Methods:**

A prospective hospital-based cross-sectional study was conducted from August to October 2017 at Debre Tabor General Hospital, Northwest Ethiopia. The Perceived Dietary Adherence Questionnaire (PDAQ) was used for dietary adherence measurement. Multivariate logistic regression was done to identify the barriers influencing dietary adherence.

**Result:**

A significant percentage (74.3%) of the study participants had poor adherence to dietary recommendations. The highest mean score was obtained for the question regarding consuming foods high in sugar with a mean 5.49 ± 1.20 times a week. On the other hand, our participants had a low consumption of fruits and vegetables and foods high in omega-3 fats with a mean of 1.84 ± 1.96 and 0.1 ± 0.62 times a week respectively. According to the survey of participants, lack of knowledge, lack of diet education, inability to afford the cost of healthy diet and poor awareness about the benefit of dietary recommendations were the most cited reasons for poor dietary adherence. In multivariate logistics regression, low level of educational status, the presence of co-morbidities, lack of previous exposure to dietary education and low monthly income were statistically significant factors associated with non-adherence.

**Conclusion:**

The rate of non-adherence to dietary recommendation among patients with T2DM was found to be high in northwest Ethiopia. Hence, providing customized health education about the potential benefit of proper dietary recommendations in controlling blood glucose is recommended. Health care providers should be proactive in promoting adherence to dietary recommendations in patients with T2DM.

## Introduction

According to the latest data from the World Health Organization (WHO), an estimated 422 million adults are living with diabetes mellitus (DM) worldwide [1]. In 2013, the International Diabetes Federation (IDF) estimated that 381 million people having diabetes [2]. This figure is expected to be almost double by 2030. Diabetes mellitus occurs throughout the world, but it is more common; especially type 2 diabetes (T2DM) in more developed countries. However, there is the greatest increase in prevalence in low- and middle-income countries where most patients will probably be from by 2030. This increasing incidence in developing countries follows the trend of urbanization and lifestyle changes, including increasingly sedentary lifestyles, less physically demanding work and the global nutrition transition, marked by increased intake of foods that are energy-dense but nutrient-poor. International Diabetes Fund estimates that 14.2 million are living with diabetes in the African region [3]. To prevent type 2 diabetes and its complications, WHO recommends that patients achieve and maintain a healthy body weight, perform a regular physical activity for at least 30 min and moderate-intensity activity on most days, eat a healthy diet, and avoid sugar and saturated fats intake and tobacco use [3]. A healthy diet helps protect against malnutrition in all its forms, as well as non-communicable diseases (NCDs), including diabetes, heart disease, stroke, and cancer [4]. The American Diabetes Association (ADA) recommends that eating fruits, vegetables, whole grains, legumes, fiber-containing foods and minimizing highly sucrose-containing foods are beneficial for secondary prevention of T2DM [5]. Failure to implement such approaches will force intensification of pharmacologic treatments or result in suboptimal glycemic control. Although dietary modification has been anticipated as the keystone of T2DM management and is usually recommended as the first step, it is considered one of the most challenging aspects of diabetes management. Regular implementation of recommended dietary practice for individuals with T2DM requires collaboration between the patient and the healthcare provider. Dietary non-adherence in people with T2DM has been identified as high in both developed and developing countries [6–8]. Factors identified as reasons for poor adherence to dietary recommendations are socioeconomic status, duration of disease, lack of diabetes knowledge, cost of healthy diet and poor communication with healthcare providers are among the most cited barriers in most of the existing data [9–12]. There is, however, limited data regarding the level of adherence and barriers to dietary recommendations in individuals with T2DM in Africa including Ethiopia. A few reports in Ethiopia and other parts of Africa suggests that patients with diabetes adherence to the dietary recommendation is generally low [13–16]. In Ethiopia, there is no dietary practice guideline and there is lack of information on the proper dietary practice for people with T2DM. Moreover, because of the paucity of evidence-based research, the health policy of Ethiopia, is still unable to give attention to this topic. Conducting and documenting such research would have a positive impact on designing and implementation of dietary practice programs for people with T2DM in Ethiopia. Therefore, this study aimed at assessing the level of dietary adherence and its barriers to dietary recommendations among people with T2DM with the representation of Northwest Ethiopia.

### Methods and settings

A prospective hospital-based cross-sectional study was conducted from August to October 2017 at Debre Tabor General Hospital, northwest Ethiopia. The hospital is located in Debre Tabor town, northwest Ethiopia which is 667 km away from the capital, Addis Ababa. The hospital is equipped with 91 beds for inpatient services and different outpatient departments including chronic illness clinics.

### Patients’ recruitment

All patients with T2DM aged > 18 years who visited the hospital for follow-up from August 1–October 30–2017 were invited to participate in the study. On the other hand, those who were critically ill and unable to participate in the interview and also those who were recently diagnosed and had a follow-up of fewer than 6 months were excluded.

### Ethical considerations

Ethical approval was obtained from the ethical review board of Debre Tabor University. Informed verbal consent was also obtained from each respondent after explaining the purpose of the study. Participant’s confidentiality was guaranteed by not recording their personal identifiers on the data collection format.

### Data quality control technique

Data collectors were trained intensively on the contents of the questionnaire, data collection methods,and ethical concerns. The questionnaires were translated into Amharic (local language) so as to maintain the unbiased response. The filled questionnaires were checked daily for completeness by the principal investigator. The reliability (psychometric property) of the tool was evaluated and demonstrated a Cronbach alpha value of 0.871. The content of the questionnaires was reviewed by senior experts of nutrition, medical doctors and clinical pharmacists.

### Data collection tool and method

The data collection tool used in this study was adopted and modified from previous studies on similar topics. The questionnaire has two major parts. Part one assessed the sociodemographic characteristics of respondents. Part two included queries about dietary adherence. The Perceived Dietary Adherence Questionnaire (PDAQ) was used for dietary adherence measurement. PDAQ is nine-item questioners which is developed in 2015 by Ghada Asaad et al. to measure patient perceptions of their dietary adherence [17]. The response is based on a seven-point Likert scale to answer the question phrased as “On how many of the last 7 days did you *….*?” (Table 1). Higher scores reflect higher adherence except for items 4 and 9, which reflect unhealthy choices (foods high in sugar or fat). For these items, higher scores reflect lower adherence, therefore, for computing a total PDAQ score, the scores for these items were inverted. Patients were classified as having good dietary adherence if they eat a healthy diet for at leaset four days in the week.

**Table 1:** Perceived Dietary Adherence Questionnaire (PDAQ) score for DM patients

Item	mean, SD
On how many of the last SEVEN DAYS have you followed a healthful eating plan?	4.9 ± 0.91
On how many of the last SEVEN DAYS did you eat the number of fruit and vegetables?	1.84 ± 1.96
On how many of the last SEVEN DAYS did you eat carbohydrate-containing foods with a low Glycemic Index? (Example: dried beans, lentils, barley, pasta, low fat dairy products)	5.21 ± 1.95
On how many of the last SEVEN DAYS did you eat foods high in sugar, such as rice, potatoes, etc.?	5.49 ± 1.20
On how many of the last SEVEN DAYS did you eat foods high in fiber such as oatmeal, high fiber cereals, and whole-grain breads?	4.01 ± 1.95
On how many of the last SEVEN DAYS did you space carbohydrates evenly throughout the day?	1.67 ± 2.01
On how many of the last SEVEN DAYS did you eat fish or other foods high in omega-3 fats?	0.1 ± 0.62
On how many of the last SEVEN DAYS did you eat foods that contained or was prepared with canola, walnut, olive, or flax oils?	4.30 ± 3.24
On how many of the last SEVEN DAYS did you eat foods high in fat (such as high fat dairy products, fatty meat, fried foods or deep-fried foods)?	4.61 ± 1.61
Over all adherence – (n, %)	
Poor	238 (74.3)
Good	82 (25.7)

### Data entry and analysis

The collected data was cleaned and entered to analyze using IBM SPSS Statistics for Windows, version 20.0. Descriptive statistics (percentage, mean and standard deviation) was used to present categorical data. Multivariate logistic regression was conducted to determine the barriers influencing dietary adherence. The results were considered to be statistically significant where *p*-value was < 0.05.

## Results

Table 2 shows the socio-demographic characteristics of participants. The mean age of the participants was 52.7 and more than half of them were males. About 43.1% of the participants had no education and only 17.8% of the participants had higher education. More than half of the study participants had had total monthly incomes of less than $ 250 and about 59.4% of them were living in a rural area. The mean duration since diagnosis of DM and duration since starting DM treatment among the study participants were 5.3 and 5.2 years respectively. About 28.1% of the participants had a family history of DM and the prevalence of comorbidities was 40.3%. A significant percentage (52.8%) of the study participants had not received education about dietary recommendation and 63.8% of the total DM patients were unable to follow doctor’s recommendation regarding diet while large number (84%) of the participants had a problem of remembering eating foods according to doctor’s advice. Additional details of the study participants are found in Table 2.

**Table 2:** Socio-demographic characteristics of the respondent

Patient characteristics and clinical data	N (%)
Total number of the study population, N	320
Sex	
Male, n (%)	168 (52.5%)
Female- n, (%)	152 (47.5%)
Age- mean, (SD)	52.69 ± 12.29
Marital status	
Single- n (%)	33 (10.3%)
Married- n, (%)	218 (68.1%)
Divorced- n, (%)	56 (17.5%)
Separated- n, (%)	12 (3.8%)
Resident	
Urban - n, (%)	190 (59.4%)
Rural - n, (%)	130 (40.6%)
Educational status	
Unable to read and write-, n, (%)	138 (43.1%)
Able to read and write -, n, (%)	64 (20%)
Primary education-, n, (%)	25 (7.8%)
Secondary education- n, (%)	35 (10.9%)
Higher education- n, (%)	57 (17.8%)
Occupational status	
Student - n, (%)	4 (1.25%)
Unemployed - n, (%)	82 (25.6%)
Government employed- n, (%)	78 (24.3%)
Self-employed- n, (%)	121 (37.9%)
Non-governmental employed- n, (%)	35 (10.9%)
Monthly income in USD	
> 250- n, (%)	43 (13.4%)
150–250- n, (%)	95 (29.7%)
< 150- n, (%)	182 (56.6%)
Duration since diagnosis of DM - mean, (SD)	5.31 ± 3.87
Duration since starting DM treatment - mean, (SD)	5.16 ± 3.77
Family history of DM	
Yes	90 (28.1%)
No	230 (71.9%)
Co-morbidity	
Yes	129 (40.3%)
No	191 (59.7%)
Physical exercise	
Yes	122 (38.1%)
No	198 (61.9%)
Previous exposure to any education regarding diet recommendation from healthcare providers	
Yes	148 (46.2)
No	172 (52.8)
Follow doctor’s recommendation regarding diet	
Yes	116 (36.2)
No	204 (63.8)
Encounter problem of remembering eating foods according to doctors advise	
Yes	269 (84.0)
No	51 (16.0)

### Perceived dietary adherence questionnaire (PDAQ) score

The mean scores of each item of **PDAQ** are shown in Table 3. The highest mean score was obtained for the question *‘*On how many of the last SEVEN DAYS did you eat foods high in sugar, such as rice, potatoes, etc.*?’* The second highest mean score was obtained for the question *‘On how many of the last SEVEN DAYS have you followed a healthful eating plan?’* participants obtained the lowest mean score, for the question *‘On how many of the last SEVEN DAYS did you eat fish or other foods high in omega-3 fats?’ and* On how many of the last SEVEN DAYS did you eat the number of fruit and vegetables?*.* A significant percentage (74.3%) of the study participants had poor adherence based on **PDAQ** while only 25.7% of the participants had good adherence towards dietary recommendations.

**Table 3:** Perceived Barriers influencing adherence to the recommended diet

Barriers	%
Lack of knowledge/lack of diet education	87
Unable to afford Cost of the recommended diet	78
Don’t believe diet can control blood glucose	67
Lack of Appetite for recommended diet	17
Unable to remember the recommended diet	57
It takes too long to cook recommended diet	21
The difficulty of adhering to the recommended diet during social or work events	39
Stress	46
Other	12

*NB: more than one answer is possible for the above questions*

### Perceived barberries influencing adherence to the recommended diet

A large number of study participants (87%) cited lack of knowledge/lack of diet education as the main barriers that hinder adherence to recommended diets. In addition to this, 78% of the participant reported that they were unable to afford the cost of the recommended diet as the reason for non-adherence. Moreover, 67% of the participants did not believe that a diet could control blood glucose. The difficulty of remembering recommended diet was cited as a reason for non-adherence by 57% of the study participants. The participants were asked whether social or work events influence their adherence to dietary recommendations and 39% of them reported that as a reason. Stress was another important barrier which was cited by 46% of the participants. In Multivariate logistic regression analysis, respondents who had no formal education were 2.6 times more likely to have poor adherence to dietary recommendations than those who had a higher education (AOR = 2.613 (95%CI, 2.021, 6.664). Regarding residency, patients who are living in a rural area were 2.4 times less adherence to their dietary recommendations for diabetes (AOR = 2.423 (95%CI 2.132–4.067). Moreover, participants with monthly income below $150 were poorly adherent to dietary recommendations (AOR = 6.781, (95%CI 2.001–9.902). The presence of co-morbidity was associated with increased non-adherence to dietary recommendations (AOR = 7.60, 95% CI, 3.29, 14.845). The occurrence of poor-adherence to dietary recommendations was 8.1 times more likely among patients who had not received any education about dietary recommendations from health care providers compared with those who had exposure to dietary education (AOR = 8.14, 95% CI,2.27–47.29). However other characteristics (age group and sex) were not significantly associated with non-adherence to dietary recommendations (Table 4).

**Table 4:** Test of association between predictive variables with level of adherence

Variables	Adherence		OR (95% CI)		P-value
	Poor (238)	Good (82)	COR	AOR	
Resident					
Urban	129	61	–	–	–
Rural	109	21	2.254 (2.015–2.922)	2.423 (2.132–4.067)	0.043^*^
Educational status					
Unable to read and write	131	7	2.241 (2.041–3.441)	2.613 (2.021, 6.664) *	0.032^*^
Primary education	16	9	0.98 (0.679–1.226)	0.643 (0.0324–1.079)	0.623
Secondary education	17	18	0.76 (0.214–2.940)	0.482 (0.09–4.371)	0.751
Higher education	57	48	–	–	–
Occupational status					
Student	2	2	–	–	–
Unemployed	70	12	2.431 (2.018, 12.790)	4.148 (2.641, 19.13) *	0.024^*^
Government employee	49	29	0.769 (0.49–2.52)	0.83 (0.55, 1.98)	0.641
Self-employed	96	25	0.62 (0.21–2.84)	1.74 (0.50, 10.66)	0.645
Non-governmental employee	21	14	0.869 (0.49–2.52)	0.93 (0.25, 1.87)	0.751
Monthly income in USD					
> 250	5	38	–	–	–
150–250	61	34	2.322 (0.845–5.799)	1.833 (0.672–5.513)	0.237
< 150	172	10	4.860 (2.003–9.484)	6.781 (2.001–9.902)	0.027^*^
Duration since diagnosis of DM					
0–5 years			–	–	–
5–10 years			0.0881 (0.0764–14.704)	0.076 (0.032–19.872)	0.075
> 10 years			2.434 (2.115–8.230)	2.814 (2.781–12.060)	0.037^*^
Duration since starting DM treatment					
0–5 years			3.74 (1.415–9.230)	5.714 (2.81–14.160)	0.07
5–10 years			0.881 (0.264–3.704)	2.626 (1.103–3.872)	0.065
> 10 years			–	–	–
Family history of DM					
Yes	29	61	–	–	–
No	209	21	1.054 (0.645–1.722)	0.823 (0.432–1.567)	0.55
Co-morbidity					
Yes	108	21	4.76 (2.55, 7.03)	7.60 (3.29, 14.845) *	0.002^*^
No	130	61	–	–	–
Physical exercise					
Yes	69	53	–	–	–
No	169	29	2.09 (0.88–4.30)	0.988 (0.0218, 1.770)	0.627
Previous exposure to any education regarding diet recommendation from healthcare providers					
Yes	79	69	–	–	–
No	191	13	5.24 (3.49–8.37)	8.14 (2.27–47.29) *	0.0001^*^

Level, * significant at 0.05 level

*AOR* Adjusted Odds Ratio, *COR* Crude Odds Ratio, *CI* Confidence Interval

## Discussion

Our study found that overall adherence levels to dietary recommendations for T2DM were poor among Ethiopian patients with T2DM, with only 25.7% of study participants following their doctor’s recommendation. This non-adherence level was comparable with studies done in other countries globally [18–21]. Another study was done on dietary practice and associated factors in Addis Ababa, Ethiopia has indicated that there was a 51.4% poor diet adherence [14] which is lower than the finding of our research. This inconsistency could be explained by the variation in the settings of the study, the difference in socioeconomics, as well as the difference in the types of foods available in the two cities.

There are established studies that examine the association between a low-carbohydrate diet and various cardiovascular risk factors in the past years. Results from a systematic review and meta-analysis of 19 randomized clinical trials showed that limiting carbohydrate intake had favorable effects on body weight, BMI, abdominal circumference, systolic blood pressure, diastolic blood pressure, triglyceride level, fasting glucose level, insulin level, HDL cholesterol level, and C-reactive protein level [22]. In addition, another meta-analysis of 13 randomized clinical trials concluded that low-carbohydrate diets in T2DM results in the improvements of glycemic control and triglyceride levels [23]. However, carbohydrate intake is higher in our study participants compared to another source of foods. The average level of carbohydrate intake for the present study participants were more than 5 times a week. This is because rice and potatoes are the staple food and primary dietary source of carbohydrates in South Gondar zone which is the study area. On the other hand, our participants had a low consumption of fruits and vegetables and foods high in omega-3 fats which is also consistent with the eating habits of the general population in Ethiopia. Seasonality of fruits and vegetables and cost could be the reason for low adherence. Although identifying barriers to dietary adherence is a complex problem and no literature has addressed these problems previously in Ethiopia, we have identified these barriers from the reports of participants and from the analysis of multivariate logistic regression. In the present study, 87% of the study participants have cited lack of knowledge/lack of diet education as the main barriers that hinder adherence to the recommended diet. The analysis of multivariate logistic regression of our study reveals that respondents who had no formal education were more likely to be poorly adherent to dietary recommendations than those who attended higher education. Moreover, poor-adherent to dietary recommendations was observed significantly in those patients who had not received any education about dietary recommendations from health care providers compared with those who had exposure to dietary education. Another important reason reported by 67% of the study participants did not believe that diet can control blood glucose. This meant that they did not know what or how much to eat, or which foods are recommended for T2DM. Therefore, improving the knowledge of diabetic patients regarding dietary recommendations with a special focus on patients with low educational level is highly important. The second most important reason reported by study participants as a reason for non-adherence to diet recommendations was the cost of the recommended diet. About 78% of the participants reported that they were unable to afford the cost of the recommended diet as the reason for non-adherence. Moreover, in the present study participants with monthly incomes below $150 were poorly adherent to dietary recommendations. Findings of the previous study also have been identified the cost of food as a barrier in non-adherence to dietary recommendation among diabetes patients [9, 24, 25]. The annual increase in the cost of healthy foods might have a negative impact on patients who were from low socioeconomic levels like Ethiopia. Therefore, providing reliable information regarding lists of low-cost healthy foods and foods that can be cheaply cooked at home may also be beneficial for patients to overcome the cost barrier for dietary adherence especially in low-income patients. In the multivariate logistic regression analysis, we have identified that the presence of co-morbidities was associated with increased non-adherence to dietary recommendations. Patients with comorbidity are often on complex medication regimens as well as complex dietary recommendations. Therefore, providing adequate information on the benefit of dietary adherence for patients with diabetes with a special focus on patients with comorbidities is an important approach. Inability to remember the recommended diet, difficulty of adhering to the recommended diet during social or work events and stress were also reported as a reason for 39–57% of patients. Less than 25% of patient-reported, lack of appetite for recommended diets and length of time to cook healthy food appears as barriers influencing adherence to recommended dietary foods for T2DM.

### Limitation of the study

This study has limitations that must be considered while interpreting the results. Firstly, self-reported data are subject to bias. There is the risk that subjects may not respond according to their true attitudes or opinions. However, this limitation is lessened by the moderately strong reliable and validated survey instrument employed in this study. Moreover, readers and researchers should be aware that the findings of the present study may not represent the population of the entire country as a source of food and other factors may vary across different geographic areas.

## Conclusion

According to our findings, non-adherence to dietary recommendations among T2DM is high as evidenced by 74.3% of the study participants reporting non-adherence. Lack of knowledge, diet education, and inability to afford the recommended diet, low monthly income, lack of previous exposure to dietary education and presence of co-morbidities were the most significant barriers responsible for non-adherence. Therefore, health professionals must become proactive in identifying and addressing these barriers and health care decision and policymakers should design effective dietary practice guideline for people with T2DM in areas where these are not available.
